# Supplementary material for: Contact pressure explains half of the abdominal aortic aneurysms wall thickness inter-study variability
Source: PLoS One. 2024 Dec 2;19(12):e0314368. doi: 10.1371/journal.pone.0314368 (PMC11611137; doi:10.1371/journal.pone.0314368)
Supplement: S2 File — This file includes detailed description of the steps in the robustness analysis used to confirm independence of the Bayesian analysis used in this study. (DOCX) [file pone.0314368.s002.docx]

# S2 Appendix Robustness analysis using alternative prior distributions

# Methods

The resulting posterior densities are significantly concentrated considering the lengths of the intervals of uniform prior distributions of the individual parameters, so we do not expect that a different choice of weakly informative prior distribution would cause a substantial change in the posterior distribution. To verify this assumption, we performed a sensitivity analysis using several alternative prior distributions:

- Prior 1: Independent Jeffreys prior distributions of the parameters $\sigma_{pop}$ and $\sigma_{pat}$ restricted to the interval [0.05, 0.4] are used instead of uniform distributions. The remaining marginal distributions are uniform.
- Prior 2: The distribution of the parameter $k$ within the interval [0, 40] is chosen so that for $c=0.38$ (posterior median for the uniform prior), the deformation $\lambda_{r}$ at pressure $p=16 kPa$ corresponding to uniaxial samples has a uniform distribution. The remaining marginal distributions are uniform.
- Prior 3: The distribution of the parameter $c$ within the interval [0, 10] is chosen so that for $k=3.35$ (posterior median for uniform prior) the deformation $\lambda_{r}$ at pressure $p=16 kPa$ has a uniform distribution. The remaining marginal distributions are uniform.
- Prior 4: The joint distribution of the parameters $c$ and $k$ is chosen as a product of the marginal densities as described in the two previous steps. The remaining marginal distributions are uniform.

# Results

The posterior distributions of the parameters $c$ and $k$ are slightly changing depending on the choice of the prior distribution, but the change in the median is negligible considering the width of the 95% credible interval. The posterior distributions of the other parameters remain virtually unchanged. However, none of the alternative prior distributions noticeably affected the resulting predictive density. Medians, credible intervals, and parameters of the lognormal approximation of the predictive density are listed in the following table.

**S2 Table 1 The variability of Medians, credible intervals, and parameters of the lognormal approximation of the predictive density depending on chosen prior distributions**

|  | $k$ (median, 95% int.) | $c$ (median, 95% int.) | predictive distribution parameters |
| --- | --- | --- | --- |
| Uniform prior | 3.35, [0.78, 11.12] | 0.38, [0.02, 1.5] | $\mu=0.85, \sigma=0.32$ |
| Prior 1 | 3.37, [0.79, 11.08] | 0.38, [0.03, 1.54] | $\mu=0.85, \sigma=0.32$ |
| Prior 2 | 2.43, [0.46, 8.1] | 0.38, [0.03, 1.34] | $\mu=0.86, \sigma=0.32$ |
| Prior 3 | 3.37, [0.79, 11.41] | 0.43, [0.03, 1.79] | $\mu=0.85, \sigma=0.32$ |
| Prior 4 | 2.4, [0.46, 8.58] | 0.43, [0.04, 1.53] | $\mu=0.85, \sigma=0.32$ |
