# Supplementary material for: Contact pressure explains half of the abdominal aortic aneurysms wall thickness inter-study variability
Source: PLoS One. 2024 Dec 2;19(12):e0314368. doi: 10.1371/journal.pone.0314368 (PMC11611137; doi:10.1371/journal.pone.0314368)
Supplement: Attachment — Submitted filename: Response to reviewers plos one V2.docx [file pone.0314368.s003.docx]

# *Response to reviewers*

The authors appreciate the valuable suggestions and comments of the reviewers, and the manuscript has been revised accordingly. Reviewers’ comments are marked in italic and our Point to point response to them is attached:

## Editor’s comments

*1. When submitting your revision, we need you to address these additional requirements. Please ensure that your manuscript meets PLOS ONE's style requirements, including those for file naming. The PLOS ONE style templates can be found at https://journals.plos.org/…pdf and https://journals.plos.org/…pdf*

Style and formatting has been adjusted to PLOS ONE's style requirements as requested.

*2. Please note that PLOS ONE has specific guidelines on code sharing for submissions in which author-generated code underpins the findings in the manuscript. In these cases, all author-generated code must be made available without restrictions upon publication of the work. Please review our guidelines at https://journals.plos.org/…ode and ensure that your code is shared in a way that follows best practice and facilitates reproducibility and reuse.*

Our computations were only partly done using sequential code generated by ourselves. The main part including model definition, MCMC simulation, and postprocessing steps with graphical outputs is now shared as requested. The other parts of computations were done interactively using various tools and commercial software and cannot be put into single code which was not our aim in this paper. We are prepared to share this detailed step by step process upon request send to email [jan.kracik@vsb.cz](mailto:jan.kracik@vsb.cz) . This is now stated in the Data Availability Statement

*3. Please amend either the abstract on the online submission form (via Edit Submission) or the abstract in the manuscript so that they are identical.*

Corrected.

*4. Please remove your figures from within your manuscript file, leaving only the individual TIFF/EPS image files, uploaded separately. These will be automatically included in the reviewers’ PDF.*

Done.

*5. Please include captions for your Supporting Information files at the end of your manuscript, and update any in-text citations to match accordingly. Please see our Supporting Information guidelines for more information: http://journals.plos.org/…ion.*

Done

Additional Editor Comments:

I congratulate the authors for working on this important topic. I believe that your manuscript will provide important data on the hemodynamics of abdominal aortic aneurysms. However, the reviewers have the following major concerns about the manuscript. Please revise your manuscript according to the suggestions and provide point-by-point answers. I wish you success.

Thank you.

## Reviewer 1

*First of all, I want to congratulate the authors for their work and for the manuscript.*

Thank you

*Reviewer comments regarding formals and language: The authors do not follow the submission guidelines (*[*https://journals.plos.org/…nes*](https://journals.plos.org/plosone/s/submission-guidelines)*) as they have the “Author summary” between the introduction and the abstract, they use abbreviations in the introduction that are not mandatory, and so on*

The formatting has been improved to follow guidelines. Abbreviations in the introduction section were reduced.

*• They do not have a uniform citation style, example: Introduction line 3 “(1)” versus line 5 “[2]”.*

Corrected.

*• Some typos remained within the manuscript, for example “gave written informed consent., and…”*

*• At some points I struggle to follow the language / the text, maybe it is due to the fact, that I am not a native speaker. Therefore, despite Grammarly has been used, I would think the manuscript could benefit from the consultation of professional language editing.*

Grammar of the revised version was checked by professional language editing service.

*Reviewer comments regarding the manuscript*

*• Following the title, the authors state that “contact pressure explains half of the abdominal aortic aneurysms wall thickness inter-study variability.*

*• The used Bayesian modelling and own, newly generated data on the aortic wall thickness, as well as already published data.*

*• The data set the authors provide contains several parameters (basic characteristics: patient number, sample number, sex, body height, body weight; descriptive parameters of the abdominal aortic aneurysm: suprarenal aortic lumen diameter, suprarenal aortic outer diameter, local ILT thickness, AAA diameter, calcification orientation; information on co-morbidities: hypertension, smoker, HLP, COPD, blood / pulse pressure).*

*• The basic problem is we know that there is a high inter- and intraindividual variance, for example demonstrated in the ascending aorta (different changes convexity versus concavity). Thus, we cannot assume that everywhere in the aneurysm the tissue has the same biomechanical properties.*

The Reviewer is absolutely correct the mechanical properties of the tissue for sure vary to some degree within each AAA and we acknowledged considering uniform mechanical properties in radial direction is a limitation of our model. Main problem is that published data says literally nothing about the mechanical response in radial direction. Vande Geest et al. 2006 and O´Leary et al. 2014 fitted their data by 2D constitutive model which cannot be even used to model response in radial direction. Munich Group (Maier 2012, Biehler et al. 2017, Biehler et al. 2018, Bruder et al. 2020) fit their data by isotropic model which for sure overestimates the radial stiffness by up to two orders, Graz Group (Tong et al. 2011, Tong et al. 2013) did not publish sample specific responses and so on. et al. Our model needs at least some information about radial stiffness, but we are currently the only group who has that data for aneurysmal tissue. When we added more parameters (like variability of c or k) to our model, we obtained very flat distributions which basically meant there is not enough information in the data to predict that. That is why we had to stick with the most general approximation assuming same radial response across the population. We hope our study will bring the attention of others to this property and other groups will provide such data. We have now reformulated the section 4.3. of the manuscript and pointed out the mismatch between initial stiffnesses obtained by various tests.

*• I was not able to find any information in the manuscript on the sampling site and how the authors controlled for “matching” sampling sites.*

The matching comes from the fact all studies reported wall samples were harvested from the anterior part of AAA which is the only part from which the sample can be taken during surgery. Samples from other parts can be taken only through autopsy. And second matching comes from our data set where in most case more than one specimen was prepared from the same sample so they are coming from adjacent regions. That gives information about intra patient variability. This is now specified in the manuscript more clearly.

*• Additionally no detailed histological data are presented. Differences in the exact degeneration can lead to different biomechanical properties, such as mucoid extracellular matrix accumulation +/- smooth muscle cell nuclei loss and so forth.*

The Reviewer is right, the histology could help to distinguish the mechanical properties. On the other hand we did not go that far on purpose and we focused only on wall thickness. Considering available information we had to chose either use only information published by others and analyze largest data set possible a obtain better information about probability of patient having very low wall thickness or work only with our data set and include more variables into our stochastic model (such as sex, calcification, HLP etc.) and accept the limitation coming from the low number of samples (therefore uncertainties would be higher) or to. Both approaches have pros and cons. In this study we focused on the former (we already have some preliminary result for the latter as well but that is consecutive work). The major problem is that such a detailed data were not published by others for individual samples so we would not be able to apply our model on their data since they simply do not exist. We feel we are too far away from predicting any mechanical properties but thickness for new patients (that is the whole point -predict it in patient who did not underwent the surgery yet, therefore we won´t have access to their histology). To underline where are we now, we would like to point out that other groups do not even report sex of the patient for each specimen on regular basis. We would be happy if our study inspires other teams to perform their mechanical testing more rigorously (reporting at least the contact pressure and patient specific data for individual samples not only summary statistics) because the mathematical models are ready, the only problem is there are no data for training those models. We have modified the manuscript to underline that.

*• The authors distinguish between presence and absence of “strong calcification”. But, they do neither define the parameter nor do they provide an adequate description of calcification. But we know that there is a big difference, especially in terms of biomechanics, intimal vs. focal medial vs. larger confluent calcifications and so forth.*

This is very good point and we had exactly the same thoughts as the Reviewer. We actually even had a model which considers the strong calcification in our data. We have a CT images for all patients and we also knew roughly the position from which the tissue was harvested so if that area contained bright spot on CT we concluded there was a calcification. Moreover, during cutting we make marks if the tissue was hard to cut (due to calcifications). So, if we found either of these conditions satisfied, we could treated the specimen as “strongly calcified” thus considering no difference between deformed and undeformed wall thickness of the specimen. So we did that work. But again we were confronted with the reality where such model could not be used for merging more data sets together as other groups do not deport that information. Consequently, the Bayesian model predicts the thickness from all samples from all groups while only 6% of our samples is marked as calcified so the overall effect on predicted population distribution of wall thickness was negligible (below 1% of undeformed thickness) Nevertheless, we mentioned the calcifications may change the wall thickness. Hopefully, others will start to report that information in the future.

Regarding the quantitative or more detailed analysis of calcification even we unfortunately did not have proper data for this (the histology). the Reviewer is perfectly right. The calcifications vary and affect the biomechanical properties. Their effect on wall thickness estimation is similar. They behave as “rocks” which cannot be compressed by pressure used so the difference between compressed and uncompressed thickness is negligible. And we also admit that we treated calcifications only qualitatively and only in our dataset, other do not provide those information for individual samples at all. When we expanded our model to incorporate effect of calcification by specific parameter the effect was below (1%) because the number of samples with known calcifications are negligible compared to total number of samples included. It is not because there were no calcified samples in other groups but because they do not report such data. Also, it is true that not knowing which samples were calcified in other data sets adds to uncertainty but there are many other factors which does the same thing (effect of sex, blood pressure, etc.) and we do not want to wait until somebody will publish data from 500samples properly measured with histology and CT images since patients with AAA need help now. Further, AAA samples are constantly harder to obtain globally as the ratio of endovascular AAA surgeries (during which the tissue harvesting is not possible) is increasing so we are even afraid that window of opportunity is closing which only strengthened our motivation for extracting the information from available data despite they are not perfect. We are not claiming this model is perfect neither it cannot be substantially improved once the more relevant data are published. On the other hand one should keep in mind the primary question we asked in this study. And that is what is the probability given patient will have very low wall thickness? And we do not think that needs to be changed anyhow.

*• Taken together, despite the very sophisticated and well-described analyses, I do not think that neither the data nor the analyses allow for the strong conclusion the manuscript draws. Without a proper description of the architecture (i.e., histopathological assessment – are the samples comparable?) and ensuring comparable sampling sites such a conclusion is for my understanding not possible. Therefore, I would recommend a major revision as I think the work the group is doing is important and can help to “move the field” by helping to increase “power” of the data and usability of data of other studies!*

The Reviewer is correct that our conclusion regarding biomechanical properties (section 4.3.) was not validated so now we did the validation and showed our constitutive model predicts comparable initial stiffnesses as study which also performed radial compression (Chuong and Fung 1984) while comparison with studies testing aneurysmal tissues in planar biaxial tests shows our stiffness is 6-8times lower. We now discuss three possible causes for that.

Histopathological assessment is certainly possible but it would require gathering new data set Moreover, we are not aware of any study where histological parameters of AAA wall would be reported for individual samples together with wall thickness measured prior sample fixation in formaldehyde. The Reviewer is certainly correct that histopathological data would help us to further decrease the observed differences between individual labs and we now advocate in the manuscript for reporting such data in the future. We only hope the Reviewer does not suggest throwing away all the data published so far and start collecting them in better way from scratch because we could not agree with that. We worked with the data available not with the data we would like to have. We expanded the limitation section to stress out our model is not intended to predict any biomechanical property but to provide the distribution of wall thickness. Our strongest conclusion is that contact pressure is a problem and it should be reported (we had to exclude all studies using caliper for wall thickness estimation so even that is certainly not universally accepted conclusion by the community). We do not see any reason why to modify such conclusion. Further we showed that even current data can be merged together and provide meaningful population distribution of wall thickness. We fully agree we (the community ) could do better in providing more data on sample specific basis and we are prepare to modify our model once such data are published.

## *Reviewer 2:*

*The present study devised a Bayesian statistical method for predicting the patient-specific AAA wall thickness by utilizing clinical data on wall thickness associated with a variety of patient-specific conditions. This research represents a substantial advancement in the field of AAA mechanics. However, significant concerns remain that require proper consideration and resolution.*

We thank the Reviewer for supportive comment.

*1. The statement “One of the most critical factors is wall thickness” is still remained to be a conjecture. Particularly, it showed that “One study found that ruptured AAAs have thicker walls(15) but others did not confirm that(16,17)” and “despite a known increase in wall strength in thinner samples(21–23)”, which is contradictory to the first statements. Thus, the first statement ought to be a verified hypothesis. This statement relies on the particular assumption that the mechanical properties are uniform and homogenous. It has been observed that the posterior wall of healthy aortas is thinner and stiffer, suggesting the possibility that a stress-mediated vascular adaptation process increases stiffness as a compensatory mechanism. Additionally, AAAs may be subjected to varying contact pressure at specific locations, such as pushing to the vertebral columns, where altered mechanical properties may result from the contact.*

Here we see we did not express ourselves clearly for which we apologize. The reviewer is right there are contradictory statements in the literature. We still think wall thickness is critical factor in AAA rupture risk analysis but it may not be as important as one would think from simply observing its effect on wall stress under assumption of homogeneous mechanical properties (just as the Reviewer correctly pointed out). This is supported from the observed inverse exponential relationship between thickness and strength which to some extend decrease the effect of wall thickness on wall stress. Nevertheless, we have eased our statement in the revised manuscript.

Unlike the Reviewer however, we are not sure what is the relation between stiffness and strength. Higher stiffness may come both from having more loadbearing fibers in the wall or having those fibers less wavy. Strength on the other hand should be related to number of fibers with the same waviness (they will straighten and rupture at the same time) We would love to investigate those things but we realized the community cannot reach agreement on even much simpler thing like what is the wall thickness in AAA.

We fully agree with the Reviewer, that various part of the AAA may be subjected to various pressure but that is not what we meant by contact pressure. The contact pressure is the pressure researchers apply on the unloaded sample during thickness measurement. And since that vary significantly from lab to lab, the outcome must vary as well. And that is systemic shift which according to our study is responsible for some 50% of intergroup variability. We already published a paper (Schwarz et al. 2023) where we propose a wall thickness measurement which will prevent this systemic shift. However, we cannot redo all the experiments ever done on AAAs so we invest our time to provide mathematical model which can take this systemic shift into account.

Finally, our aim is to estimate unloaded wall thickness and once that is known the finite element models can easily account for the fact the vertebral column is touching certain part of the wall.

*2. “To do this, the mentioned constitutive model was used to simultaneously fit all available biaxial responses of aneurysmal tissue published in (29) to obtain a mean response.” It is unclear why neither uniaxial nor biaxial experiments were conducted in the present study. It should be possible to validate whether this 3D model is comparable to the HGO model, given that the previous HGO models were based on only 2D measurements and it is uncertain whether the 3D HGO model results in having ‘unique’ sets of model parameters with all 3-D stretches. It is doubtful whether biaxial responses from references can be explicitly extrapolated from this radial test in the absence of validation.*

This is an excellent comment! The Reviewer is right, the uniaxial and biaxial tests could be in principle performed and we did them back in 2012-2016. Unfortunately, we later discovered malfunction of one of our strain gauges which corrupted most of our data. That is why our group published only one study (Polzer et al. 2013) where biaxial tests on few samples (measured with older but properly working strain gauges were used)are performed and we rather relied on data of others than fabricating what we think would be true responses of the samples.

The Reviewer also brought up good point regarding validity of HGO (or any other) model fitted on biaxial tests in radial direction. We elaborate on this more in the revised manuscript. First, we estimated the initial circumferential and axial stiffnesses in planar equibiaxial test and compared the data with available values from literature. It can be seen from Table 3 that initial stiffness derived from our model (fitted on radial compression data) is in good agreement with the only other study we found which tested aortic samples radially and provided constitutive description of it (Chuong and Fung 1984). The independent aneurysmal data fitted on planar biaxial tests shows 6-8times higher initial stiffnesses. We provided three hypotheses why. i) the inability of mentioned constitutive models correctly predict behavior in radial direction, ii) issues in wall thickness measurements (which scales the stiffness) iii) our assumption of uniform mechanical properties in radial direction.

*3. All the AAA tissue samples were only obtained from the anterior side, and it can be a critical limitation for predicting overall thickness and rupture analysis.*

That is certainly true, and we now elaborate on this topic more and mention this as a limitation as well. Problem is, that obtaining samples from other than anterior parts of AAA is rare and typically possible for autopsies only (ref 16,17,19) (with their own limitations)-typically the group around prof. Raghavan has such results. Unfortunately, we could not include their data since they still rely on operator dependent wall thickness estimation via caliper, thus the contact pressure is unknown for each sample.

*4. Please clarify why very different contact pressure values (pc_uni vs. pc_biax) were used for the contact pressure tests.*

We thank the Reviewer for this comment. It is due to using the same measuring tool (thickness measured via thickness gauge and two glasses (see Polzer et al. 2015 for more information). Thus, the applied force was constant in both cases, yet the uniaxial samples had 16x smaller surface area, resulting in 16x higher contact pressure. This is now specified in the manuscript.

*5. It needs to define the deviatoric part in Eq. (2). Since B is FF^t, eigen values are squared from F. So why lambda_r is not squared?*

We are thankful for this comment. The eq.2 was actually entirely wrong (only in manuscript though, we used it correctly in the code). It is now corrected.

*5. “ referencing a declination of each family of fibers from the circumferential direction." Specify the unit (radian?).*

Corrected.

*6. Correct “ = (| −2⁄3 )”*

Corrected.

*7. Define “FPK”*

Done.
