# Supplementary material for: Contact pressure explains half of the abdominal aortic aneurysms wall thickness inter-study variability
Source: PLoS One. 2024 Dec 2;19(12):e0314368. doi: 10.1371/journal.pone.0314368 (PMC11611137; doi:10.1371/journal.pone.0314368)
Supplement: Attachment — Submitted filename: Response to reviewers plos one R2V1.docx [file pone.0314368.s004.docx]

# *Response to reviewers*

The authors appreciate the valuable suggestions and comments of the reviewers, and the manuscript has been revised accordingly. Reviewers’ comments are marked in italic and our Point to point response to them is attached:

## Editor’s comments

*Dear Authors,*

*I must say that we are pleased that you have revised and uploaded your manuscript in a proper way. Your manuscript has been reviewed by three referees, one previous and two new. There were concerns especially about the statistical methodology and hence one of the reviewers was a biostatistician and their suggestions are below and I am confident that your manuscript will reach a higher level of quality in line with these suggestions. Please have the final version of your revised manuscript edited by a native English speaking expert. I wish you success.*

We thank the editor for kind comment. Regarding the English, we had the native speaker even before but we chose another one in order to address persisting concerns about grammar which we are not able to address by ourselves since none of us is native speaker. The other comments are discussed below.

*Reviewer #1:*

*Dear Stanislav Polzer and colleagues,*

*Thank you very much for the revision of the manuscript! I want to congratulate you for the work you put in the manuscript! And thank you for giving background information with your answers to the reviewer’s comments.*

We are glad we answered your questions.

*Just two very minor points, I personally think you can easily and swiftly include:*

*Point (A):During the revision, you included the following sentence: “This is because it is not possible to extract tissue from other parts of the AAA without threatening the patient so the only possibility to obtain data from other parts is to harvest AAAs from autopsies (19,35).”*

*I know that this “thesis” is published in some instances. But there are conditions, in which resection even of the posterior parts are done, for example, in the rare mycotic aneurysm, where it can be (rarely) necessary to remove the infected tissue. Thus, the statement, that this is not possible, is for my understanding not correct. I would recommend the following: “**This is because in the routine tissue from other parts of AAA is not resected.” With such a general statement you are “safe”, I personally think.*

We changed the sentence as suggested.

*Point (B):During the course of the abdominal aorta from the diaphragm to the bifurcation the aortic wall changes the “wall properties” in terms it becomes more and more a “muscular vessel”. Therefore, especially in long aneurysm, anterior does not equal anterior. In your answer to the reviewer comments you explained that you “knew roughly the position from which the tissue was harvested”. Could you please just provide this localization somewhere in the methods section, for example, samples of approximately at height of the renal arteries? In case I did not capture the information during the re-review, I apologize for this unnecessary comment!*

The position of harvest was almost exclusively in the middle between renal arteries and aortic bifurcation. We have mentioned that in the chapter 2.1. now. We (biomechanics) did not want samples close to bifurcation where the geometry is complex and it is not possible to prepare flat specimens out of it (for biaxial testing which was the original motivation why the samples were harvested. On the other hand, surgeons were careful not to cut the specimen too close to renal arteries since the tissue there is need for sewing the artificial artery.

*After these two minor points are addressed, I think the manuscript should be ready to be accepted.*

*Thank you very much for your important work and looking forward to read your paper! Best wishes!*

We thank the Reviewer for positive recommendation.

*Reviewer #3:*

*This is an incredibly interesting paper, but unfortunately, the authors could not unlock its full potential mainly due to the drawbacks of the analyzed studies/datasets, which resulted in significant limitations such as possibly much higher inter- and intra-patient variability. Nevertheless, I would like to express my utmost respect to the authors, as this is one of those studies that raises an important questions and can spur further research. In addition, the authors provided detailed characteristics of each patient, which certainly can aid other teams in conducting similar studies.*

We thank the Reviewer for supporting comment.

*1) When I was invited to review this manuscript, only the abstract was available to me. Given the nature of this study, I believe this paper needs to be reviewed by a biostatistician. I lack sufficient expertise to provide an opinion on some aspects of this paper, but I can and will review other parts that are more closely related to cardiovascular medicine. That being said, I have very little to add beyond what has already been mentioned by previous reviewers.*

*2) "professional language editing service" mentioned by the authors doesn't seem to be very professional, as there are still many errors in the manuscript, some of which are very basic. For example, "our results is based" (Discussion, 1st paragraph; incorrect noun-verb agreement), "vena cava inferior" (Introduction, 2nd paragraph; incorrect word order), etc. Apart from grammatical errors, many sentences just don't sound natural. Please, have a native speaker revise your paper.*

We apologize for that. The revised manuscript was checked by other grammar editor, so we hope it is ok now. Unfortunately, this is beyond our expertise as none of us is native speaker so we had to trust the grammar editor we chose.

*3) I read the authors’ response to Reviewer 1 about the “strongly calcified” column in the supplementary file. I don’t think that the authors’ response is sufficient since virtually any calcification (not just “strong calcification”) fits their description. The description needs to be more elaborate and clear and must be provided in the supplementary file as well.*

We are sorry, if our response did not satisfy the Reviewer, but we cannot do any better. That is the definition we used back in the days we collected the samples so we cannot change it now. Since the Reviewer thinks the definition is misleading, the only thing we can do now is to erase that information from the supplementary material completely. That was done.

*4) The authors need to be more elaborate and clear. Their responses to the reviewers are rather detailed and easy to understand, whereas the changes applied in the manuscript lack cohesion and appear much more confusing (e.g. the passage about calcifications), mostly due to poor English.*

We are sorry. The presence of calcifications was erased form the manuscript and it remained only as the limitation. The English was corrected by another editor so hopefully, it is ok now. The major problem however is that those who are experts in grammar are not necessarily experts in soft tissue biomechanics.

*5) Any inclusion/exclusion criteria? What aneurysms were included in this study? Symptomatic? Asymptomatic? Ruptured?*

Only asymptomatic AAAs were included in this study. It is now mentioned in the revised manuscript

*6) Fig. 6 needs to be fixed. There are no spaces between words, making it difficult to read. Also, please upload figures in the correct order next time.*

We are sorry, the Figure 6 was fixed as requested.

*Reviewer #4: There are some comments about the equations and validation method.*

*In equation 1, the subscript k is not written (k1 and k2). On the other hand, these symbols are not fully explained.*

Corrected. However, k is intentionally used without subscript.

*The two symbols Ψiso and Ψaniso are undefined.*

Corrected.

*Recheck the definition of symbols in equation 1 in lines 113-115.*

*The bold symbols for tensors and vectors were missing.*

Corrected.

*In equation 3, I think Wiso and Waniso should be replaced by Ψiso and Ψaniso. Also, in formula 2, the subscript λ is not correct.*

Symbols for strain energy density functions and subscripts for lambda were corrected.

*In line 135, why is λ_r considered in the range of 0.61 to 1?*

In order to cover the expected range of radial deformations during in-vivo deformations which is tensile in axial and circumferential direction but compressive in radial direction. This is now stated in the manuscript.

*In Table 2, the scale of the first column (from the left) is not written correctly. Also, this table is not referred to in the text.*

Thank you. Scale was corrected and the reference to that table was added into the text.

*It is suggested to perform the sensitivity analysis for the robustness of the results compared to the selection of other non-informative and vague prior distributions.*

The resulting posterior densities are significantly concentrated considering the lengths of the intervals of uniform prior distributions of the individual parameters, so we do not expect that a different choice of weakly informative prior distribution would cause a substantial change in the posterior distribution. To verify this assumption, we performed a sensitivity analysis using four alternative prior distributions. The results are now summarized in **S2** **Appendix.** The priors slightly affected the posterior distributions of the parameters $c$ and $k$. The posterior distributions of the other parameters remained virtually unchanged. However, none of the alternative prior distributions noticeably affected the resulting predictive density of the undeformed thickness. A note has been added to Section 2.3.

*In addition to the methods mentioned in Section 2.5, why have simulations or artificial data not been used to assess validation of the model?*

The correctness of the calculation of the joint distribution of the statistics $M_{k}, S_{k}$ was repeatedly validated by comparison with artificial data samples. A note has been added to Section 2.5.

The resulting predictive distribution of unloaded thicknesses is also validated using a set of simulated data samples (grey histogram in Figure 7). The simulated data are then compared with the unloaded thicknesses and their estimates (blue histogram in Figure 7). This is already commented in Section 3.5.

*Considering the almost big difference in the rupture risk of AAA in men and women, why the same parameter was considered for both?*

It was simply due to the fact that any other group did not provide information about sex of the sample donor. Therefore, we could not use that. We are in the similar situation as in case of calcifications which was commented by other reviewers previously. Yes, we agree it would be useful to add those parameters but that would basically double number of parameters, yet we are currently the only group who provide information about sex of the sample donor so no improvement on prediction would be gained.

*Is it possible to investigate the effect of variables such as age and smoking, etc. on the rupture risk of AAA?*

This study is purely aimed at prediction of AAA wall thickness, not AAA rupture risk. In order to estimate rupture risk, one need to know distribution of thickness, strength (which also strongly depends on thickness) and peak wall stress (which depends on wall thickness as well). That is why we started with the first step which is obtaining wall thickness distribution. After that, we could continue for strength distribution and stress distribution.

Regarding other parameters, yes. Proposed model can be easily modified to add any parameter one may think about such as smoking, or age, but without a data, no relevant posterior prediction can be made. Again, it is excellent point, and we would love to do it but we are facing the reality where other groups rarely even report sample based information about thickness. We hope our paper will initiate the debate about changing it and people will start reporting more sample-specific information, but we (the biomechanical community) are not there yet.
